# How to respond to resistiveness towards assistive technologies among persons with dementia

**DOI:** 10.1007/s11019-017-9816-8

**Published:** 2017-12-06

**Authors:** Anders Nordgren

**Affiliations:** Centre for Applied Ethics, Linköping University, 581 83 Linköping, Sweden

**Keywords:** Assistive technologies, Autonomy, Best interest, Dementia, Nudging, Resistiveness

## Abstract

It is a common experience among care professionals that persons with dementia often say ‘no’ to conventional caring measures such as taking medication, eating or having a shower. This tendency to say ‘no’ may also concern the use of assistive technologies such as fall detectors, mobile safety alarms, Internet for social contact and robots. This paper provides practical recommendations for care professionals in home health care and social care about how to respond to such resistiveness towards assistive technologies. Apart from the option of accepting the ‘no’, it discusses a number of methods for influencing persons with dementia in order to overcome the ‘no’. These methods range from various non-coercive measures—including nudging—to coercion. It is argued that while conventional caring measures like those mentioned are essential for survival, health or hygiene, assistive technologies are commonly merely potentially beneficial supplements. With this in mind, it is concluded that care professionals should be more restrictive in using methods of influence involving some degree of pressure regarding assistive technologies than regarding conventional caring measures.

## Introduction

Assistive technologies are becoming increasingly important in home health care and social care. Examples of technical devices are fall detectors, mobile safety alarms with GPS positioning and Internet for social contact with care professionals or relatives. In not too distant a future robots may also become significant.

However, these technologies raise serious issues about ethical values like autonomy, privacy and justice (Hofmann 2013; Nordgren 2013). In particular this is the case when technologies of this kind are used in care for persons with dementia (Perry et al. 2009; Nuffield Council of Bioethics 2009, pp. 97–99; Niemeijer et al. 2010; McLean 2011; Landau and Werner 2012; Novitzky et al. 2015; Gordijn and ten Have 2016). This use may sometimes create special problems. As has been shown in several empirical studies, it is a common experience among care professionals and informal carers that persons with dementia often say ‘no’ to conventional caring measures such as taking medication, eating or having a shower (Mahoney et al. 1999; Volicer and Vongxaiburana 2009; Tranvåg et al. 2013; Fauth et al. 2015). This tendency to say ‘no’ may also concern assistive technologies (Niemeijer et al. 2015). The aim of this paper is to present proposals for how to respond to such resistiveness in an ethically acceptable way.

The paper consists of two major parts. In the first part, I discuss resistiveness towards conventional caring measures in home care among persons with dementia. I indicate when to accept a ‘no’ to such measures and when to influence in order to overcome the ‘no’. I also present a number of possible methods for how to proceed in influencing persons with dementia and indicate when it is justified to use these methods. This first part provides the necessary background to the second part in which I turn to resistiveness towards assistive technologies. In this second part, I investigate to what extent the proposals in the first part regarding conventional caring measures are applicable to assistive technologies. The main focus in both parts is on the decision-making of care professionals working in elderly peoples’ homes, although the proposals may hold also for care professionals in nursing homes and informal carers.

## Resistiveness towards conventional caring measures

### Dementia and resistiveness

Dementia is a broad category of progressive neurodegenerative disorders. Its most common form is Alzheimer’s disease. Dementia ranges from mild to severe with no clear boundaries. Persons with dementia suffer from memory loss and thinking difficulties, which increases with time. The behaviour and mood can be very different from prior to dementia. Persons with dementia have more or less reduced competence, i.e. ability to understand and decide. A person with mild dementia can be rather competent, while a person in a more advanced stage can be rather incompetent in many respects. There is often a variation in competence over time for one and the same person. The lack of competence is often task-specific. The person may be quite competent regarding some things and incompetent regarding others (Nuffield Council of Bioethics 2009, pp. 4–6, 8–12, 23–26; Fellows 1998; Holm 2001).

It is fairly common that persons with dementia say ‘no’ to conventional caring measures. In the literature this is called resistiveness, non-compliance or rejection of care (Ishii et al. 2012). The fundamental reason for resistiveness may be that the persons with dementia are more or less cognitively impaired. They may not understand the question about a proposed caring measure. They suffer from memory loss and do not remember how things usually are. Their mind is disordered in various ways. Something inside may tell persons with dementia that it is good to say ‘no’ to most questions. It is just the easiest thing to do. The care professionals may speak too fast. Persons with dementia may be incapable to ingest all information. The ‘no’ may depend on how the question is phrased (Alzheimer’s Reading Room 2012).

However, there may also be other reasons for the ‘no’ than that the person is cognitively impaired. The ‘no’ may be a reaction to elderspeak, i.e. the tendency of some care professionals to speak as if the person were a small child. It may be a reaction to the caring person pushing too hard to persuade. The person with dementia may have a strong sense of privacy and resist what is felt to be intrusions of privacy. The person may be worried, for example regarding meeting a dentist or moving to a nursing home with a lot of unknown people (Alzheimer’s Reading Room 2012; Williams et al. 2009).

So, in some cases the ‘no’ is a response due to lack of competence. Persons with severe dementia may not have understood the question or simply respond in an irrational way. But in other cases a ‘no’ is a fairly competent ‘no’. Persons with mild dementia have understood the question sufficiently well and dislike the suggestion for quite rational reasons.

### A realistic conception of autonomy

Beauchamp and Childress point out that “competence in decision-making is closely connected to autonomous decision-making” (Beauchamp and Childress 2013, p. 114). However, in order to understand and ethically assess the autonomy of persons with dementia we need a realistic conception of autonomy. In this regard, Agich makes a useful distinction between ideal and actual autonomy. Ideal autonomy is the traditional liberal conception that focuses on independence and non-interference of fully competent agents. Agich criticizes this conception when applied to elderly people who may lead very dependent lives. Actual autonomy of the elderly is linked to the individual’s sense of identity rather than independence and non-interference. What is important is to be able to retain identity even when facing dependencies. This sense of identity constitutes a basis for decision-making also for persons who are very dependent on caregivers and others (Agich 1990, 2003; cf. Nuffield Council of Bioethics 2009, pp. 26–28).

Given this realistic account of autonomy, persons with mild dementia may qualify as persons with a capacity for autonomy. They have some competence to understand and decide although they may be very dependent on others for their daily life. Even persons with more severe dementia may qualify to the extent they still retain a sense of identity. They may retain such a sense even if their behaviour diverges substantially from prior to dementia. In this identity-focused sense they continue to have some limited autonomy, even though they are seriously lacking in competence regarding some specific tasks. However, in the most advanced stages persons with dementia may have lost also their sense of identity. They may have no memories of their previous lives or only mere glimpses without connection (Nuffield Council of Bioethics 2009, pp. 4–6, 9–12, 23–26).

Agich’s realistic conception of autonomy has implications for the obligation of care professionals to respect autonomy. Agich states:

A contextual account is wanted that attends to the phenomenon of actual rather than ideal autonomy. The implications for long-term care of this turn to actual autonomy are important. Respect for autonomy cannot mean that caregivers are primarily and absolutely precluded from influencing the decisions of elders. To be exposed to influence as such is not to be enslaved (Agich 1990, p. 14).

So, Agich proposes not only a realistic conception of autonomy (as a capacity) but also, by implication, a realistic conception of respect for autonomy (as an obligation). A realistic conception of respect for the autonomy of elders does not preclude influencing their decisions. To respect autonomy is to assist the elderly in retaining their sense of identity rather than to abstain from interfering with their lives.

This holds also for elderly persons with dementia. Sometimes it may be ethically justified to influence them when they say ‘no’ to caring measures that care professionals and close family members deem to be in their best interest. But when more exactly should care professionals influence them in order to overcome the ‘no’ and on what grounds? And when should they just accept the ‘no’? These are issues to which we now turn.

### Accept or influence?

Whether or not care professionals should influence persons with dementia to overcome their ‘no’ to conventional caring measures depend in part on the degree of dementia. The competences of persons with dementia lie on a continuum with two extremes. At the one extreme there are persons with very mild dementia who have only very minor reductions of competence regarding specific tasks, at the other extreme persons in the most advanced stage of dementia with major reductions of competence and who may have lost their legal right to autonomy by court order. This variation in competence among persons with dementia may sometimes make decisions on whether or not to influence them very difficult. As Holm stresses:

In the end the conclusion must therefore be that no amount of rules will ever be able to relieve the care giver of his or her obligation to personally assess the desires and decisions of the demented and possibly incompetent patients and ethically choose which to respect and which to counteract (Holm 2001).

However, even if the personal assessment by care professionals is indispensable and no rules can ever relieve them from the obligation to make such assessments, this does not mean that rules or at least some ‘points to consider’ cannot be practically useful. Even if it is necessary to make decisions on a case-by-case basis, this does not exclude that certain ‘points to consider’ can guide care professionals in their daily work. I will provide some proposals in this regard.

The default option when persons with dementia say ‘no’ to a conventional caring measure should be to accept the ‘no’. Perhaps the measure is not necessary right now or within the next day or days. However, regarding conventional caring measures such as taking medication, eating or having a shower, it might at some point become justified to influence persons with dementia in order to overcome the ‘no’—sometimes even with some degree of pressure—because these measures are essential for survival, health or hygiene. It is not possible to wait any longer and there are no alternatives. In this situation it is vital that care professionals and close family members reach a common agreement that it would be in the best interest of the person with dementia to influence. It is also vital that they agree on how to proceed in influencing. The earlier care professionals and close family members agree on these issues the better for the person with dementia.

In making decisions about influencing a person with dementia, care professionals and close family members should take into consideration also the person’s preferences prior to dementia. This is how Nuffield Council of Bioethics stresses the importance of balancing past and present preferences of persons with dementia:

This [Codes of Practice] guidance should emphasise that neither past nor present can automatically take precedence, but that the relative strength of the person’s wishes, the degree of importance of the decision, and the amount of distress being caused should all be important factors to consider (Nuffield Council of Bioethics 2009, p. 83).

In decision-making regarding conventional caring measures such as taking medication, eating or having a shower, care professionals have good reasons to believe that the present ‘no’ of the persons with dementia to such measures is in conflict with their past preferences as expressed in their daily living prior to dementia and also when their dementia was in its early stages. At that time they did take their medication, eat and have showers. Such past preferences should be taken into account when deciding what is in the persons’ best interest, even if they are not decisive on their own. In deciding whether or not to influence persons with dementia, care professionals should take into consideration and balance these persons’ past and present preferences, the views of close family members on what is in the persons’ best interest, and their own professional views on what is essential for good care.

In cases of resistiveness towards conventional caring measures such as taking medication, eating or having a shower, the difficult problem for care professionals is often not to decide whether to influence the person with dementia in order to overcome the ‘no’. The problem is rather how to proceed in influencing, i.e. what methods to use (see further below). However, at other times decision-making on whether or not to influence can be rather difficult, for example in cases where persons with dementia say ‘no’ to going to the dentist or moving to a nursing home. It can be even more difficult in cases such as when persons prior to dementia have expressed the wish that if they develop dementia they would not want any life-prolonging medical treatment and at present when they suffer from dementia they seem quite content but are unable to articulate any preferences regarding life-prolonging treatment (Nuffield Council of Bioethics 2009, p. 81).

### Possible methods of influence

If care professionals and close family members agree that they can no longer accept the ‘no’ of persons with dementia regarding conventional caring measures that are essential for survival, health or hygiene, the next step is to take a stand on what method of influence should be used.

Practical recommendations on how to influence patients with psychiatric problems can be found in the literature (Widdershoven and Berghmans 2007). However, the main focus has been on the ethics of coercive methods. The ethics of more subtle forms of influence has not received adequate attention (Blumenthal-Barby et al. 2013).

Practical recommendations on how to influence persons with dementia more specifically have also been proposed (Nuffield Council of Bioethics 2009, p. 83; Tranvåg et al. 2013; Bolmsjö 2006; Smebye et al. 2016). However, a systematic presentation of practical recommendations on how to respond to resistiveness among persons with dementia is by and large lacking. Some practical suggestions can be found on websites of various patient organizations (Help for Alzheimer’s Families 2017; Alzheimer’s Reading Room 2012; Caring.com 2017), but these are neither presented in a systematic way nor put into a theoretical framework.

Moreover, the practical recommendations regarding resistiveness among persons with dementia do not explicitly include the concept of nudging. This concept has recently received attention in other areas, originally in behavioural economics (Thaler and Sunstein 2008), but also in medical ethics (Cohen 2013; Saghai 2013). However, it has not yet been explicitly applied to resistiveness among persons with dementia, although some recommendations by patient organizations include practical measures that can be considered as examples of nudging.

With this in mind, I propose as a starting-point for reflection the following list of possible methods for influencing persons with dementia who say ‘no’ to conventional caring measures (I do not claim the list to be complete). In the list I present brief explanations of the methods and give some examples. Further below I discuss under what circumstances it could be ethically justified to use the methods. The methods are ordered from various non-coercive interventions to coercion.

1. *Personalized information* (for the reason of obtaining a ‘yes’) This means that the information is not given in a standardized fashion but truly adapted to the level of understanding of the patient. Elderspeak is avoided.
2. *Rephrasing the question* For example, instead of “Do you want to…?” the care professional could say “You have always liked to do this, haven’t you?” or “This is fine, isn’t it?” or “This is just a test, don’t you want to try?”
3. *Rational persuasion* The care professional provides various arguments why the caring measure is good. For example, “It is important that you eat, because…” or “It is important that you have a shower, because…” The care professional may also refer to the persons’ views prior to dementia and point out that they have always liked eating food and having showers.
4. *Nudging* Nudging means influencing behaviour by changing the choice architecture. The options are the same but they are presented in a different way (Cohen 2013). For example, if the caring measure is to go out and get some fresh air, instead of saying “Let’s go out” the care professional could say “It’s a lovely day out. Let’s go out”, or “It’s a lovely day out. Let’s go out in the garden to look at the roses”. A close family member could do it the same way or perhaps turn to this option: “It’s a lovely day out. Let’s go out in the garden to a look at the roses. I need your advice to prune them properly” (Help for Alzheimer’s Families 2017). In all these cases the two options of choice are to go out or to stay indoors, but the option of going out is presented differently. Another way of nudging is to present going out as default: “We always help old people to go out in the sun. It is very appreciated”.
5. *Incentive* An incentive is an additional positive experience provided as a reward after the caring measure has been carried out. The reward is unrelated to the caring measure in the sense that it is not a direct consequence of the caring measure as such. It is an additional benefit. For example, if the caring measure is to help the patient to have a shower, an option is to state “Afterwards we will eat ice cream”. Another option is to say “You will get something good to eat but first you will have a shower” (Alzheimer’s Reading Room 2012).
6. *Appeal to authority* Elderly persons with dementia may have a strong inclination of respect for doctors. Given this, an option is to appeal to doctors’ traditional authority and plainly state “This is doctor’s orders” or ask the question “Don’t you want to do as the doctor says?” (Help for Alzheimer’s Families 2017).
7. *Deception* If the person with dementia refuses to take medication—and this medication is deemed by the care professional to be essential for the person’s health—a deceptive measure could be to crumble the pills and hide the pieces in the food. This measure comes close to coercion, since it intentionally deceives the person in his best interest. The person with dementia is not aware of what has been done and has not given consent.
8. *Coercion* If a person in an advanced stage of dementia resists a caring measure that is essential for survival, health or hygiene, some degree of coercion might be a last option. In distinction to deception the person may in this case be aware of what is going on. An example could be a fall accident resulting in a fracture. If the person refuses to go to the hospital for clinical examination and treatment, a last option could be to use coercion.

### When to use the methods

These methods represent only possibilities. The difficult question is under which circumstances it could be ethically justified to use them.

Methods 1, 2, and 3 have the common feature that the influence of the care professional on the person with dementia can be described as fully non-controlling (Saghai 2013). An appeal is made only to the rational deliberation of the individual. No pressure of any kind is exerted. Autonomy is fully respected. The methods only represent refined ways of obtaining informed consent. These methods may be sufficient when persons who say ‘no’ to the caring measure have only mild dementia. These persons are competent enough and can still make decisions that are in their own best interest.

Method 4 (nudging) relies on non-deliberative or incompletely deliberative processes rather than rational persuasion. It does not change the options that the individual faces—to accept or not accept the caring measure—but changes the choice architecture. Nudges exert their influence by organizing the context of decision in a new way (Saghai 2013). Nudges in this sense have been suggested as a way of preserving a respect for individual autonomy but at the same time influence people to make what is considered good decisions. As mentioned above, nudging was originally proposed in behavioural economics but has recently also been discussed in medical ethics. From the perspective of behavioural economics, a nudge is defined by Thaler and Sunstein as “any aspect of the choice architecture that alters people’s behaviour in a predictable way without forbidding any options or significantly changing their economic incentives” (Thaler and Sunstein 2008, p. 6). A classic example of nudging is when the manager of a coffee house places healthy food, for example fruit, at the beginning of the queue and the unhealthy at the end with the effect that guests are more likely to buy the healthy food. Another example is the US Medicare where generic medication is given as default but the brand-name drug is a secondary option (Saghai 2013). Nudging is a matter of interfering “so as to preempt the gap between patient preference and medical recommendation” (Cohen 2013). It respects individual autonomy but still represents a kind of weak paternalism. Thaler and Sunstein call it ‘libertarian paternalism’ (Thaler and Sunstein 2008). Nudging is substantially non-controlling but may still influence (Saghai 2013). It can be an ethically justified method of influencing a person with dementia, since it still respects the person’s autonomy. However, even if nudging would be ethically justified questions can be raised concerning its effectiveness. To what extent can nudging be effective when it comes to influencing persons whose minds are more or less distorted? The effectiveness is at least indicated by the fact that the examples of nudging given above actually are from the website of an Alzheimer’s organization (Help for Alzheimer’s Families 2017). Even though the term ‘nudging’ is not used, the organization actually recommends such measures based on the collective experiences within the organization.

In method 5 an appeal is made to positive experiences that will follow if the individual accepts the caring measure. It does not stress the valuable effects of the caring measure itself, but promises a reward afterwards as a means of influencing the individual to accept the caring measure. This type of influence may range from substantially non-controlling to substantially controlling, depending on the details (Saghai 2013). The incentive is substantially non-controlling when it is tempting but not too much. Such incentives might be ethically justified as a way of influencing persons with mild or moderate dementia. The incentive is substantially controlling when the persons are given offers they can hardly refuse. This might be justified in some cases regarding persons with severe dementia.

The question is how far nudging and providing incentives can get us. Care professionals might reach a point where more pressure becomes necessary. We see this in method 6. The appeal to the authority of the doctor represents a slightly more paternalistic approach than nudging and providing incentives. It is somewhat negatively charged compared to these other two approaches, which evoke positive feelings. It stresses that the doctor knows the best in matters of health and might involve a more or less hidden indication that the persons with dementia are to be blamed if they do not follow doctor’s orders. This appeal to authority can be characterized as substantially controlling, but not fully controlling. It can probably be ethically justified in some cases of more advanced forms of dementia.

Method 7 (deception) and method 8 (coercion) are even more paternalistic than methods 4, 5, and 6. These options will probably not be justified when the person has mild dementia and retains some competence. Deception is fully controlling. In this case the person with dementia is not aware of what is actually going on. Coercion is also a measure that is fully controlling, but in this case the person with dementia may be aware of what is happening. If applied at all, coercion could probably be ethically justified as a last option only regarding the most advanced forms of dementia and regarding caring measures that are very urgent and essential for survival, health and hygiene. Moreover, the coercion should be humane, i.e. the coercion should be as minimal as possible and be in the best interest of the person. The example above of the fall accident might be a case when it is ethically justified to use such humane coercion.

## Resistiveness towards assistive technologies

In the previous section, I argued that it is sometimes ethically justified to influence persons with dementia in order to overcome the ‘no’ to conventional caring measures offered in home health care and social care. I also discussed a number of methods for how to proceed. I will now investigate to what extent my proposals hold also for the use of assistive technologies in this type of care.

### Reasons for resistiveness towards assistive technologies

Let us start with the issue why persons with dementia may say ‘no’ to assistive technologies. Partly, they may do so for the same reasons as those mentioned above regarding conventional caring measures, for example, problems of ingesting information, a reaction to elderspeak or feelings of intrusion of privacy. However, when it comes to assistive technologies, there may be also other reasons for saying ‘no’.

One special reason may be reduced task-specific competence regarding particular assistive technologies (Perry et al. 2009; Ganyo et al. 2011). Persons with dementia may say ‘no’ because they do not understand how the technology works (at a layperson’s level of understanding) and how it will affect their daily living. They may have special difficulties in understanding how to use the technology. Regarding some technical devices it may be necessary to do something, for example press a button on a mobile safety alarm. However, regarding some other technologies they need not to do anything for the technology to work. An example is fall sensors.

Another reason is that persons with dementia may be particularly worried about assistive technologies, since they have not met this technology before. Persons with dementia may have difficulties of coping with new things in general, but unfamiliar technologies might cause particular anxiety.

A third reason is due to the fact that assistive technologies are quite controversial in society in contrast to conventional caring measures. Some persons with dementia might have been very critical towards assistive technologies—or towards technology in general—prior to developing dementia and continue to be critical also when they have developed this disorder.

### Challenges in using assistive technologies for persons with dementia

Why use assistive technologies for persons with dementia? The justification for a large-scale introduction of such technologies for an ageing population in general is commonly stated to be care for the health and quality of life of the patients. By using these technologies it could be possible for elderly people to live at home longer and avoid, or at least postpone, the need to move to a nursing home. By devices for monitoring heart conditions or diabetes attached to the body or implanted into the body the number of hospital visits and hospitalisations could be reduced. Instant information from fall detectors could make it possible for care professionals to help persons in fall accidents quicker than otherwise. Getting lost when taking a walk outdoors could be avoided by mobile safety alarms with GPS positioning. The Internet could help elderly persons to stay in touch with their children and friends but also with care professionals (Nordgren 2013).

However, driving forces behind the development of these technologies are not only concerns for health and quality of life but also technological ambitions, commercial opportunities and the wish among high-level decision-makers for cost-effectiveness in the use of limited health care resources (OECD Health Policy Studies 2010). This raises the issue to what extent this development truly is in the best interest of patients with dementia (Gordijn and ten Have 2016; Novitzky et al. 2015). At the very least these driving forces are contextual factors to take into consideration when assessing the ethical justification of using the technologies.

With this in mind, it becomes particularly important to investigate the point of using assistive technologies for persons with dementia, especially if some of them say ‘no’ to using them. Why should care professionals influence persons with dementia to use these technologies? To what extent can these technologies be beneficial and to what extent can they be harmful? Let me give two examples of special importance, one example of a potential benefit and one of a potential harm.

A potential benefit is related to the special importance of being able to continue to live at home when you are a person with dementia. Above I referred to Agich’s realistic conception of autonomy, ‘actual autonomy’. On this conception, autonomy concerns retaining the individual’s sense of identity rather than aiming for independence and non-interference (Agich 1990). Now, home is the place where the person’s sense of identity is likely to be best retained (for other aspects of the value of home for persons with dementia, see Dekkers 2011). The close relationship of home and identity has been pointed out by Chaudhury and Rowles:

It is now widely accepted that home provides a sense of identity, a locus of security, and a point of centering and orientation in relation to a chaotic world beyond the threshold (Chaudhury and Rowles 2005, p. 3)

But living at home as a person with dementia—rather than moving to a nursing home—may require assistance by health care and social care professionals. However, given the limited resources of health care and social care, care professionals cannot be present around the clock, and this might not be what the person with dementia wants, either. So, assistive technologies might be useful supplements to conventional care by making it possible for persons with dementia to continue to live at home (Nuffield Council of Bioethics 2009, pp. 97–99; Smebye et al. 2016).

A potential harm of assistive technologies is related to the special need of many persons with dementia to experience personal contact. As Agich points out:

Elders must be treated as individuals, as unique persons with identifiable personal histories so far this is possible. Even when such identifications are difficult to assess, as in cases of severe memory deficits associated with Alzheimer’s disease, patients frequently respond, albeit minimally and in deficient ways, to direct contact with caregivers and others (Agich 1990, p. 16–17).

This need for direct personal contact also among persons with severe dementia speaks against reducing the number of face-to-face meetings with care professionals by remote monitoring technologies for reasons of cost-effectiveness. Cost-effectiveness is certainly ethically relevant at a policy-making level. Assistive technologies are expected to reduce costs, for example by reducing the number of visits to general practitioners and health care centres, reducing the number of hospitalisations and reducing the time for hospitalisations (Finkelstein et al. 2006; OECD Health Policy Studies 2010). However, at the level of care for individual patients individual needs must be given priority. For many elderly persons the need for personal contact with care professionals is of vital importance. For some patients face-to-face meetings with care professionals may be their only opportunity for personal contact, since all their friends have already died. Assistive technologies should be considered supplements to personal meetings with care professionals rather than substitutes (Nordgren 2014). Persons with dementia should not be pressed to use technologies for reasons unrelated to their particular individual needs.

So, assistive technologies may support the wish of persons with dementia to retain a sense of identity by living at home rather than moving to a nursing home. However, they may also be ethically problematic when used to replace personal meetings with care professionals by reference to cost-effectiveness. In making decisions on whether or not to use assistive technologies for persons with dementia, this potential benefit and this potential harm should be taken into serious consideration.

### Accept or influence?

Given these challenges, should care professionals accept the ‘no’ of persons with dementia to assistive technologies or should they influence them in order to overcome the ‘no’? As in the case of conventional caring measures, the default option should be to accept the ‘no’. However, while in the case of conventional caring measures—such as taking medication, eating or having a shower—it might at some point become justified to influence the person, this is not to the same extent the case regarding assistive technologies. There are at least two reasons for this.

One reason is that in contrast to conventional caring measures assistive technologies are commonly not essential for survival, health or hygiene, but merely potentially beneficial supplements. They may make it possible for persons with dementia to continue to live at home for a longer time rather than moving to a nursing home. In this regard fall detectors, mobile safety alarms and Internet for social contact could be useful. But commonly assistive technologies are not essential for health in the way conventional caring measures are. However, there are exceptions. An example is the traditional safety alarm for indoor use that a person wears around the neck or wrist. In case of a fall the person may press a button on the alarm and avoid serious harm by getting help quickly.

Another reason for being more restrictive in exerting pressure regarding assistive technologies than regarding conventional caring measures has to do with the economic context. To the extent assistive technologies are not essential for survival, health or hygiene but merely potentially beneficial supplements, it becomes particularly problematic to exert pressure if the technological devices are to be paid for by the persons with dementia themselves (or their families). This would hardly be in line with respecting the autonomy of these persons. However, if the devices are to be paid for by public health services or community services (perhaps at a small fee) it might be acceptable to exert some pressure in order to overcome the ‘no’, although it is vital that the assistive technologies are assessed to be truly beneficial supplements for the persons with dementia and not used as substitutes for personal encounters with care professionals.

With this in mind, care professionals should generally be more restrictive in influencing persons with dementia regarding assistive technologies than regarding conventional caring measures. This does not preclude that in some cases some pressure can be exerted also regarding assistive technologies.

### Possible methods of influence

Some recommendations in the literature on methods of influence focus on assistive technologies in general (Niemeijer et al. 2015), others on particular devices (Landau and Werner 2012). However, these recommendations are neither sufficiently specific nor sufficiently systematic. Let us therefore tentatively apply the possible methods of influence discussed previously in relation to resistiveness towards conventional caring measures. Here are some examples of how this could be done. Further below I discuss when and to what extent it is ethically justified to use the various methods.

1. *Personalized information* The information about the technical device—its use and impact on everyday living—is adapted to the level of understanding of the patient.
2. *Rephrasing the question* For example, the care professional may say “This is just a test, don’t you want to try this new mobile safety alarm?”
3. *Rational persuasion* The care professional stimulates deliberation and tries to convince the person by providing various arguments for the benefits of using the device. For example, the care professional could say “It is important that you use this mobile safety alarm, because then you can go outdoors, and if you don’t find your way home, you can get help quickly” or “It is important that we install these fall sensors, because then we can come and help you rapidly if you fall on the floor”.
4. *Nudging* Nudging in this context could mean to present the benefits of the technical device in a vivid but non-deliberative or not completely deliberative manner. For example, the care professional can stress the contrast between using and not using the device by first showing a video about what happens if the device is used and then showing what happens if the device is not used (or the other way around). In the case of fall detectors, persons with dementia will get help rapidly if they have fallen on the floor, but without fall detectors they may have to wait very long for help. In the case of a mobile safety alarm with GPS positioning, persons with dementia can take a walk outdoors on their own and still be safe, but without it they might get lost and it might be very difficult to find them. In the case of Internet, they can very easily have contact with children and grandchildren, but without using it they will sit alone for long periods of time. Another way of nudging is to present the device as default (if this is the case in this organisation): “We install fall sensors as a standard measure for older people who have a tendency to fall”, “We always provide mobile safety alarms to people who have problems finding their way home after a walk”, “We always give older people who live alone the opportunity to have contact with their children via this kind of screen”.
5. *Incentive* For example, regarding Internet for social contact an option is to say “If you use this screen, your children would be very happy and send you a box of chocolate!”
6. *Appeal to authority* Also with regard to assistive technologies it might be possible to appeal to the doctors’ authority and stress “This is doctor’s orders” or ask “Don’t you want to do as the doctor says?”
7. *Deception* An example could be to install a new floor but abstain from telling that it has inbuilt fall sensors. Persons with dementia are just told that they will get a new and nicer floor.
8. *Coercion* A possible example is GPS tagging to prevent that persons with severe dementia are harmed by getting lost.

### When to use the methods

Now, the question arises to what extent it is ethically justified to use these methods of influence. In order to discuss this issue in a way that takes seriously possible ethically relevant differences among assistive technologies, let us take a closer look at the three technologies referred to as examples in the list: fall detectors, mobile safety alarms and the Internet.

It is common that persons with dementia fall frequently. By using fall detectors that instantly transmit the information to the care professionals, the person with dementia may get help more rapidly than otherwise. Moreover, some harm due to prolonged undetected fall accidents may be prevented, for example hypothermia and dehydration. So, there seem to be good reasons to influence persons with dementia to have fall detectors installed in their homes. Two types of fall detectors exist, namely fall sensors—for example floor sensors or motion sensors—and video monitoring (Ganyo et al. 2011). The former might seem less privacy intrusive than the latter. They just register, for example, that the person with dementia has fallen from the bed to the floor and transmit this information to care professionals. Video monitoring, on the other hand, may register in detail what the person looks like and also everything else that the person does in addition to falling. However, to the extent the picture in made less detailed, for example by blurring it, also video monitoring might be acceptable from a privacy point of view. Since fall detectors can be truly beneficial the first four methods of influence could be used and probably also methods 5 and 6. In some cases where the persons with dementia fall very frequently it might even be justified to use methods 7 and 8. What may make decisions about exerting pressure difficult concerning fall detectors is that there often exists a good alternative in terms of traditional safety alarms. However, in cases of severe dementia the persons may not have capacity to press the button on the alarm. In those cases fall detectors can be essential for health. Decisions on these issues need to be based on case-by-case assessments.

Mobile safety alarms with GPS positioning make it possible for persons with dementia to walk outdoors on their own (Landau et al. 2011; Landau and Werner 2012). This is certainly perceived by many to be of great value. If the persons do not find their way home they can press a button on the device and care professionals can track them via the GPS system. In trying to influence persons with dementia to use this device the first four methods—and possibly also methods 5 and 6—could be used. However, if persons with dementia still do not want to use GPS devices, would it under any circumstances be ethically justified to use deception (method 7) or coercion (method 8)? In cases where persons with dementia have a dangerous tendency to just leave home without anyone knowing where they go and they often get lost, an option could be to hide GPS devices in their clothes or shoes without informing them. However, I doubt that such deceptive or coerced GPS tagging could be justified. There seems to exist a good alternative, namely that they are assisted in their outdoor walking by care professionals or relatives. However, decisions on these matters can be very delicate and require a balancing from case to case.

In discussing mobile safety alarms let me also mention one more technical option, namely to add geographic fencing to the alarm. This technology could be even more problematic from an ethical point of view than ordinary mobile safety alarms. Geographic fencing means that if persons with dementia walk outside a predetermined geographical area, care professionals or close family members will be informed. This might be beneficial in cases where persons with dementia have a strong tendency to have problems finding their way home after a walk. However, geographic fencing might be perceived as privacy intrusive by the person with dementia, since if the person walks outside the geographic fence, for example to the pub, care professionals or close family members would know and the person might not want that. This could be a reason to not influence the person by deception or coercion to use such a technology.

Social contact via the Internet can be of great value for persons with dementia who feel lonely (Cotten et al. 2013). The contact can take place via an ordinary computer screen, but also via a bigger screen covering part of the wall, creating a feeling of being almost present in the homes of children or grandchildren. Other devices are communication robots (a kind of “Skype on wheels”) following persons with dementia when they walk around in their apartment or house. Social contact can slow down the process of dementia, and the Internet could be very useful in this regard. However, these devices require active participation by the persons with dementia. There can be no meaningful social contact via the Internet if they do not want to communicate. So, using the first five methods could be justified. Appeal to authority (method 6) will probably not work, since a positive attitude is necessary for true communication, and feeling pressure from an authority like a doctor might not promote such a positive attitude. For the same reason coercion (method 8) will probably not work, either. True communication cannot be based on coercion. Deception (method 7) is non-applicable, since care professionals cannot deceive a person with dementia to communicate via the Internet.

In the list of methods of influence I didn’t give any examples regarding robots. The reason is that robots are still to a large extent something for the future. There are different kinds of robots. I have already mentioned communication robots. Other types are companion robots, such as robot seals or robot cats, and robots that assist the person with dementia in eating or finding the way in the house (Sharkey and Sharkey 2012; Sorell and Draper 2014). However, robots might be problematic for persons with dementia in other ways than the other technical devices. Robots may cause anxiety because persons with dementia may not understand their true nature, i.e. that they are merely complicated machines. To the extent an assistive robot is considered to be in the best interest of a person with dementia the first four methods of influence—and maybe even methods 5 and 6—might be used. Deception (method 7) would not work, at least not for the near future, since the robot prototypes developed so far are not similar enough to human beings in their looks and behaviour. However, when they become similar enough, deceiving persons with dementia would still hardy be ethically justified, because there exists a better alternative, namely to provide more assistance by care professionals. For the same reason coercion (method 8) should probably not be used. Care professionals should avoid deception and coercion, because robots seem neither essential for survival, health or hygiene, nor beneficial enough supplements to conventional caring measures for these methods to be justified.

## Conclusion

In this paper I have developed my argument in two steps. The first step concerns whether or not care professionals should influence persons with dementia who say ‘no’ conventional caring measures such as taking medication, eating or having a shower. I suggest that the default option should be to accept the ‘no’, but argue that at some point it might be justified to influence the person with dementia in order to overcome the ‘no’ (sometimes even with some degree of pressure), because these measures are essential for survival, health or hygiene.

This first step provides the necessary background to the second step, in which I investigate to what extent the proposals regarding conventional caring measures are applicable to the use of assistive technologies. I argue that while conventional caring measures like those mentioned are essential for survival, health or hygiene, assistive technologies are commonly merely potentially beneficial supplements to these conventional caring measures. With this in mind, I conclude that care professionals should be more restrictive in using methods of influence involving some degree of pressure regarding assistive technologies than regarding conventional caring measures.
